# Multiple Neural Oscillators and Muscle Feedback Are Required for the Intestinal Fed State Motor Program

**DOI:** 10.1371/journal.pone.0019597

**Published:** 2011-05-05

**Authors:** Jordan D. Chambers, Joel C. Bornstein, Evan A. Thomas

**Affiliations:** 1 Department of Physiology, The University of Melbourne, Parkville, Australia; 2 Florey Neuroscience Institutes, Parkville, Australia; 3 Centre for Neuroscience, The University of Melbourne, Parkville, Australia; Mount Sinai School of Medicine, United States of America

## Abstract

After a meal, the gastrointestinal tract exhibits a set of behaviours known as the fed state. A major feature of the fed state is a little understood motor pattern known as segmentation, which is essential for digestion and nutrient absorption. Segmentation manifests as rhythmic local constrictions that do not propagate along the intestine. In guinea-pig jejunum *in vitro* segmentation constrictions occur in short bursts together with other motor patterns in episodes of activity lasting 40–60 s and separated by quiescent episodes lasting 40–200 s. This activity is induced by luminal nutrients and abolished by blocking activity in the enteric nervous system (ENS). We investigated the enteric circuits that regulate segmentation focusing on a central feature of the ENS: a recurrent excitatory network of intrinsic sensory neurons (ISNs) which are characterized by prolonged after-hyperpolarizing potentials (AHPs) following their action potentials. We first examined the effects of depressing AHPs with blockers of the underlying channels (TRAM-34 and clotrimazole) on motor patterns induced in guinea-pig jejunum, *in vitro*, by luminal decanoic acid. Contractile episode durations increased markedly, but the frequency and number of constrictions within segmenting bursts and quiescent period durations were unaffected. We used these observations to develop a computational model of activity in ISNs, excitatory and inhibitory motor neurons and the muscle. The model predicted that: i) feedback to ISNs from contractions in the circular muscle is required to produce alternating activity and quiescence with the right durations; ii) transmission from ISNs to excitatory motor neurons is via fast excitatory synaptic potentials (EPSPs) and to inhibitory motor neurons via slow EPSPs. We conclude that two rhythm generators regulate segmentation: one drives contractions within segmentation bursts, the other the occurrence of bursts. The latter depends on AHPs in ISNs and feedback to these neurons from contraction of the circular muscle.

## Introduction

The major functions of the gastrointestinal (GI) tract are the digestion of food, absorption of nutrients and excretion of waste. The smooth muscle of the GI tract uses several complex motor patterns to perform these tasks. These motor patterns are controlled by the enteric nervous system (ENS), which is located within the intestinal wall, and can generate, and switch between, these motor patterns independently of the central nervous system [1]. After a meal, the duodenum and jejunum exhibit a complex set of contractile patterns, collectively known as the *fed state*, which facilitates digestion and absorption [2]. *In vivo* studies have identified three broad contractile patterns in the fed state: non-propagating, propagating and retrogradely propagating contractions [3]. The relative proportions of these three patterns and the switches between them determine the rate of transport of the contents along the intestine and hence the efficacy of the digestive and absorptive processes [4], [5], [6]. They depend at least in part on the nutrients within the intestinal content [5], [7], [8]. However, the mechanisms by which food and the derived nutrients drive this critical motor function and the underlying neural activity remain ill-defined at best. The connectivity of the ENS is reasonably well established, but the dynamic interactions between subclasses of neurons and how these drive different contractile patterns is poorly understood.

In the upper small intestine, duodenum and jejunum, of the guinea-pig, luminal decanoic acid and some amino acids induce fed state-like motor activity *in vitro* [9], [10]. This activity consists of stationary contractions, short length (1–2 cm) slowly propagating contractions and whole length propagating (or propulsive) contractions. Both the stationary contractions and short length contractions are unique to segmentation, with the properties of the short length contractions making them likely to be considered non-propagating in *in vivo* studies. They occur in rhythmic bursts grouped together with occasional propulsive contractions into active episodes that can last from 40 to 60 seconds. The active episodes are separated by quiescent episodes lasting 40 to 200 seconds. This rhythmic behaviour appears to be controlled by ENS *in vitro* because it is blocked by agents that interfere with neural transmission [10] and does not require smooth muscle pacemaker potentials (slow waves) [9]. Slow waves are difficult to record in the isolated guinea-pig intestine, despite the presence of interstitial cells of Cajal (ICC), but are much more prominent in other species like mice and humans. However, even in species where slow waves are prominent, the ENS plays a major role in regulating motor patterns seen in the fed state [11]. The virtual absence of a contribution to these patterns in guinea-pigs by ICCs makes the guinea-pig jejunum an ideal preparation to investigate the neural involvement in nutrient induced motility patterns.

Much is known about the enteric neural circuitry of guinea-pig small intestine (for reviews see [12], [13], [14]). Motility is largely controlled by the network of myenteric neurons lying between the longitudinal and circular muscle layers [15]. These neurons include intrinsic sensory neurons (ISNs), interneurons, excitatory motor neurons and inhibitory motor neurons. Most ISNs send at least one axonal projection to the mucosa [16] which can be activated antidromically by serotonin acting on 5-HT_3_ receptors on the terminal membrane [17]. ISNs are active during the fed-state motor pattern because blocking 5-HT_3_ receptors at the level of the mucosa abolishes segmenting contractions [18] and application of some amino acids to the mucosa activates ISNs via mucosal serotonin release [19]. Furthermore, recent direct measurements of serotonin release from guinea-pig intestinal mucosa suggest that this can result from smooth muscle contraction [20].

The ISNs have excitatory outputs to feed forward interneuron networks that run either orally or anally along the gut wall [13], [21]. The orally directed pathway couples to the muscle via excitatory motor neurons [22]. The anally directed pathway couples to the muscle via inhibitory motor neurons [23]. The ISNs also connect monosynaptically to local motor neurons [19]. Transmission from the ISNs to the motor neurons is via fast excitatory synaptic potentials (EPSPs, lasting about 50 ms) mediated by ligand gated ion channels and via qualitatively distinct slow EPSPs (lasting 3–120 s) mediated via G-proteins coupled to second messenger pathways (for review see [14]). Transmission between ISNs is via slow EPSPs [14]. Previously, we suggested that the rhythm generator responsible for contractions within a burst is located in the ISN network [24], but how these bursts are organised into longer temporal patterns is unknown. Activity within the ISN network is organised by interactions between slow EPSPs and the inhibitory effects of a prominent after-hyperpolarising potential (AHP) following action potentials in these neurons [25], [26], [27]. Inhibitory input is also seen in ISNs [28], but its role in GI motor programs is unknown.

We examined the role of the ISN network using an *in vitro* model of nutrient induced segmentation [9], [10], [29], [30]. Fed state motor activity was induced in isolated guinea-pig jejunum by luminal decanoic acid, a robust nutrient stimulus whose effects are mimicked by other luminal nutrients [9], [30]. The role of ISNs in motor activity induced by decanoic acid was investigated pharmacologically by examining the effects of two compounds (TRAM34, clotrimazole) that depress AHPs [31]. We also examined the effects of antagonists of 5-HT_1A_ receptors, as these receptors may be involved in inhibitory synaptic potentials in ISNs [28].

To understand the *in vitro* results, we built a lumped computer model of ISNs and enteric motor neurons. The model included feedback from muscle contraction that mimicked the time course of contraction induced mucosal serotonin release, fast and slow excitatory synaptic transmission between neurons and damping of ISN activity due to AHPs. The model reproduced the qualitative results of the *in vitro* experiments and made several predictions about the circuitry involved in this complex motor pattern.

The goal of this study was to identify the roles of ISNs in motor patterns of the fed state, notably segmentation. Our video-imaging showed that there are two distinct motor pattern generators regulating segmentation in guinea-pig jejunum *in vitro*: one determines contraction frequency within bursts, while the other determines the burst frequency. The modelling indicated that the properties of this second pattern generator are determined by AHPs within the ISNs and by feedback from the muscle, making the muscle itself part of the circuit.

## Results

### Pharmacology experiments

#### Contraction counts

**Figure 1:**
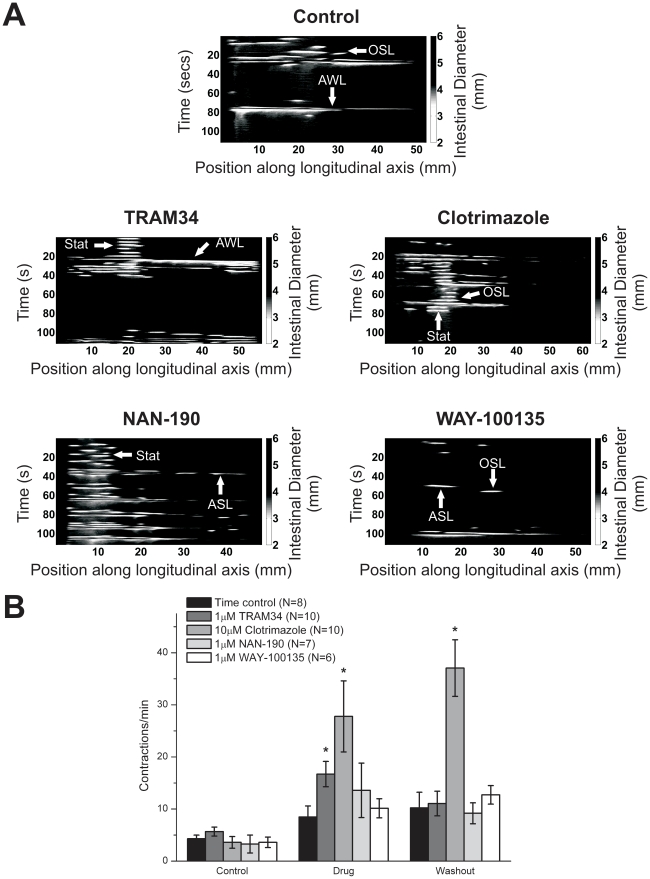
Effect of drugs on overall contractile activity. *A)* Example spatiotemporal maps highlight the different types of contractions and different number of contractions observed. The light colour represents contractions. The arrows point to contractions were AWL represents anally propagating whole-length contractions, ASL represents anally propagating short-length contractions, OSL represents orally propagating short-length contractions and stat represents stationary contractions, *B)* Total contraction counts during 20-minute video recordings for control and for each pharmacological agent. Asterisks indicate a significant difference in the number of contractions between drug and control recordings (determined by unpaired t-tests, p<0.05).

Video recordings were processed into spatiotemporal maps to allow classification and quantification of contractions (Figure 1A). To suppress the AHP, 1 µM TRAM34 or 10 µM clotrimazole were used, as these concentrations suppress the I_K_ current underlying the slow AHP [31]; while 1 µM NAN-190 and 1 µM WAY-100135 were used because these concentrations are effective in blocking inhibitory synaptic transmission to enteric neurons [28], [32]. Once the drugs had washed into the bath for 20 minutes, a significant increase in the number of contractions was observed for TRAM34 and clotrimazole, but not NAN-190 or WAY-100135. The effects of TRAM34 washed out within 20 minutes, whereas the effects of clotrimazole did not wash out after 60 minutes (Figure 1B and Table S1).

**Figure 2:**
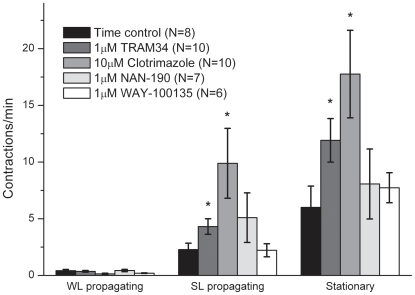
Effects of pharmacological agents on contraction counts. Contraction counts for whole length propagating contractions (WL), short length propagating contractions (SL) and stationary contractions during the control recording or 20 minutes after introducing the drug to the bath superfusate. Asterisks indicate a significant difference in the number of contractions observed between control recording and drug (determined by unpaired t-tests, p<0.05).

Contractions were further classified as whole-length propagating contractions (WL), short-length propagating (SL) contractions or stationary contractions, with orally or anally propagating contractions counted separately (Figure 2 and Tables S2, S3, S4). None of the agents altered the total number of WL contractions, which largely consisted of anally propagating WL contractions. Orally propagating WL contractions were only rarely observed. TRAM34 and clotrimazole both increased the number of SL contractions, but NAN-190 and WAY-100135 had no effect on SL contractions. TRAM34 increased the number of anally propagating SL contractions, but had no effect on orally propagating SL contractions. However, clotrimazole increased the numbers of both anally propagating and orally propagating SL contractions. TRAM34 and clotrimazole both increased the number of stationary contractions observed, whereas NAN-190 and WAY-100135 had no effect on stationary contractions.

In summary, agents that depress the AHP in ISNs significantly increased the numbers of SL propagating contractions and stationary contractions without affecting the whole-length propagating contractions. Agents that may block IPSPs in some ISNs did not significantly alter the numbers of any type of contractions.

#### Contraction properties are not affected by blocking inhibitory currents

The length (or distance of propagation along the longitudinal axis) and speed of contractions was measured (Table S5 and Table S6). None of the agents significantly affected the length of muscle recruited, the speed of propagation of anally directed WL propagating contractions, the length and speed of anally and orally directed SL propagating contractions or the length of stationary contractions. The exception is that clotrimazole significantly decreased the length of anally directed SL contractions. Orally directed WL propagating contractions were rare and no analysis of their characteristics was possible.

In summary, the characteristics of the different types of contractions were unaffected by blocking either I_K_ channels or 5-HT_1A_ receptors.

#### Blocking the AHP increases the frequency of isolated bursts

**Figure 3:**
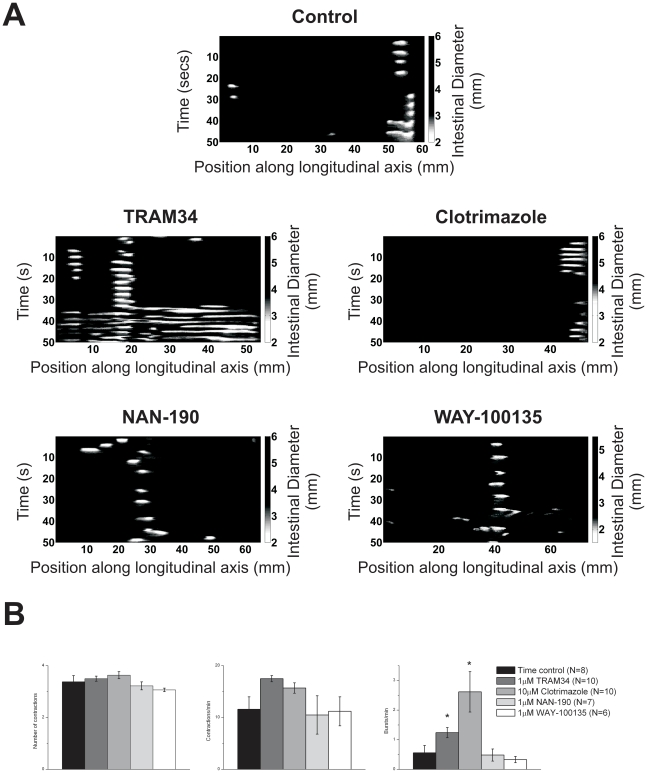
Properties of isolated bursts of contractions. *A)* Example spatiotemporal maps highlighting isolated bursts of stationary and SL contractions. The lighter grey represents contractions. Histograms of *B)* the average number of contractions in each burst, *C)* the frequency at which contractions occurred within each burst, and *D)* the number of bursts observed. The asterisks indicate a significant change in the number of contractions between control and drug recordings (determined by unpaired t-tests, p<0.05).

During segmentation, stationary and SL propagating contractions are often observed in bursts of several contractions at the same location. Figure 3A shows examples of isolated bursts of contractions for each pharmacological agent. The criteria defining isolated bursts are described in the methods. For each isolated burst identified, the number (Figure 3B) and frequency of contractions within the burst (Figure 3C) were measured. The frequency of isolated bursts was also determined (Figure 3D).

None of the pharmacological agents affected the number of contractions within an isolated burst nor did they affect the frequency of contractions within an isolated burst. However, both TRAM34 and clotrimazole significantly increased the frequency of isolated bursts, while NAN-190 and WAY-100135 did not (Table S7 in the supporting information for quantitation).

In summary, none of the pharmacological agents affected the properties of isolated bursts of activity. However, agents that depress AHPs in ISNs increased the frequency of isolated bursts.

#### Blocking the AHP increases the duration of active episodes, but not quiescent episodes

**Figure 4:**
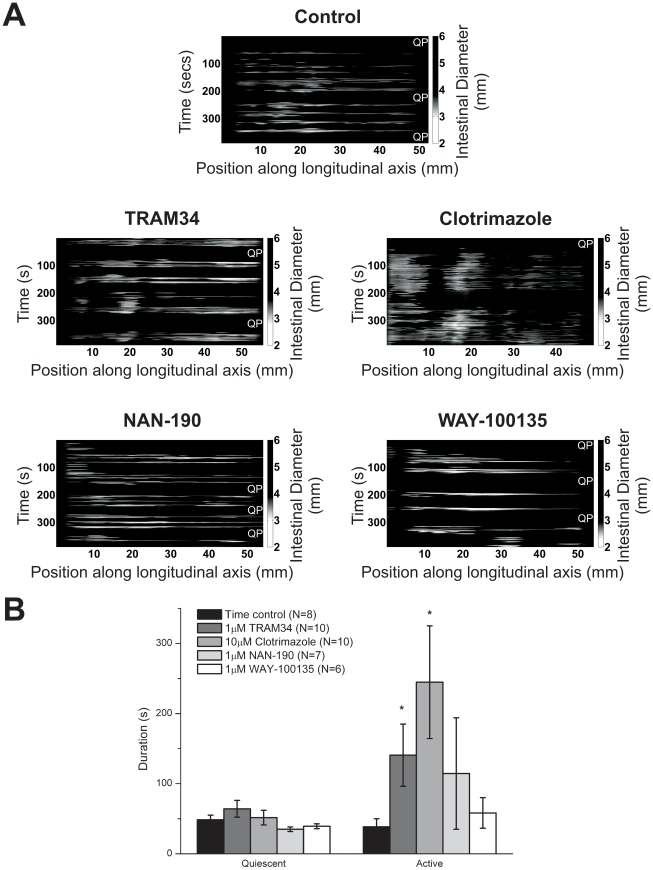
Effects of drugs on active and quiescent episodes. *A)* Example spatiotemporal maps showing active and quiescent episodes. Lighter colours represent contractions. Quiescent episodes are indicated by QP. *B)* Histograms of active and quiescent episode durations, before and during drug application. The asterisks indicate significant differences between the control and drug recordings (determined by unpaired t-tests, p<0.05). See Table S8 in the supporting information for quantitation.

During segmentation, single contractions and whole-length propagating contractions are all mixed in together during episodes of activity that are separated by periods of quiescence. Figure 4A shows examples of spatiotemporal maps that highlight active and quiescent episodes. For purposes of quantitation, a quiescent episode was defined as 20 seconds or longer without contractions and active episodes were defined as the periods between quiescent episodes.

The durations of activity and quiescent episodes are plotted in Figure 4B. None of the agents affected the duration of the quiescent episodes. However, TRAM34 and clotrimazole significantly increased the duration of the active episodes, while NAN-190 and WAY-100135 had no effect.

In summary, depressing the AHP in ISNs significantly increased the duration of active episodes without affecting the duration of the quiescent episodes. Blocking IPSPs in ISNs had no effect on the duration of the active episodes or the quiescent episodes.

### A model of bursting contractile activity

An abstract model was developed to understand how depressing the AHP in ISNs can increase the duration of active episodes without affecting the duration of quiescent episodes. The model described activity in ISNs, excitatory and inhibitory motor neurons and in the circular muscle. When drive to the excitatory motor neurons was higher than drive to the inhibitory motor neurons, there would be activity in the circular muscle. This activity was interpreted as an active episode, consisting of a number of different contractions and contraction types over this period of time. It is beyond the scope of this model to produce individual contractions. Instead, this model produced periods of time where individual contractions could readily occur due to the differences in activity in the excitatory motor neurons and inhibitory motor neurons. Similarly, when activity in the inhibitory motor neurons was higher than activity in the excitatory motor neurons, there was no contractile activity in the circular muscle and this was interpreted as a quiescent episode.

#### The control response

The ‘control response’ model is described in the methods and supplemental material and reproduces the temporal characteristics of the switch between quiescent and active periods of segmentation in response to decanoic acid. Many aspects of synaptic transmission between different classes of neurons remain uncertain and indeed the purpose of the model is to explore the consequences of different transmission dynamics. As described below, some parameters had little effect on output, but the model was very sensitive to changes in other parameters leading to physiological predictions. The model consists of the major neural elements on the ENS and incorporates excitatory feedback resulting from muscle contraction with the time course of the excitatory feedback matching contraction induced release of serotonin from the mucosa. However, it should be noted that it is the feedback, not the serotonin release, that is modelled. The aim of the model was to understand the temporal organization of segmentation, time courses of components in the model were made as realistic as possible.

**Figure 5:**
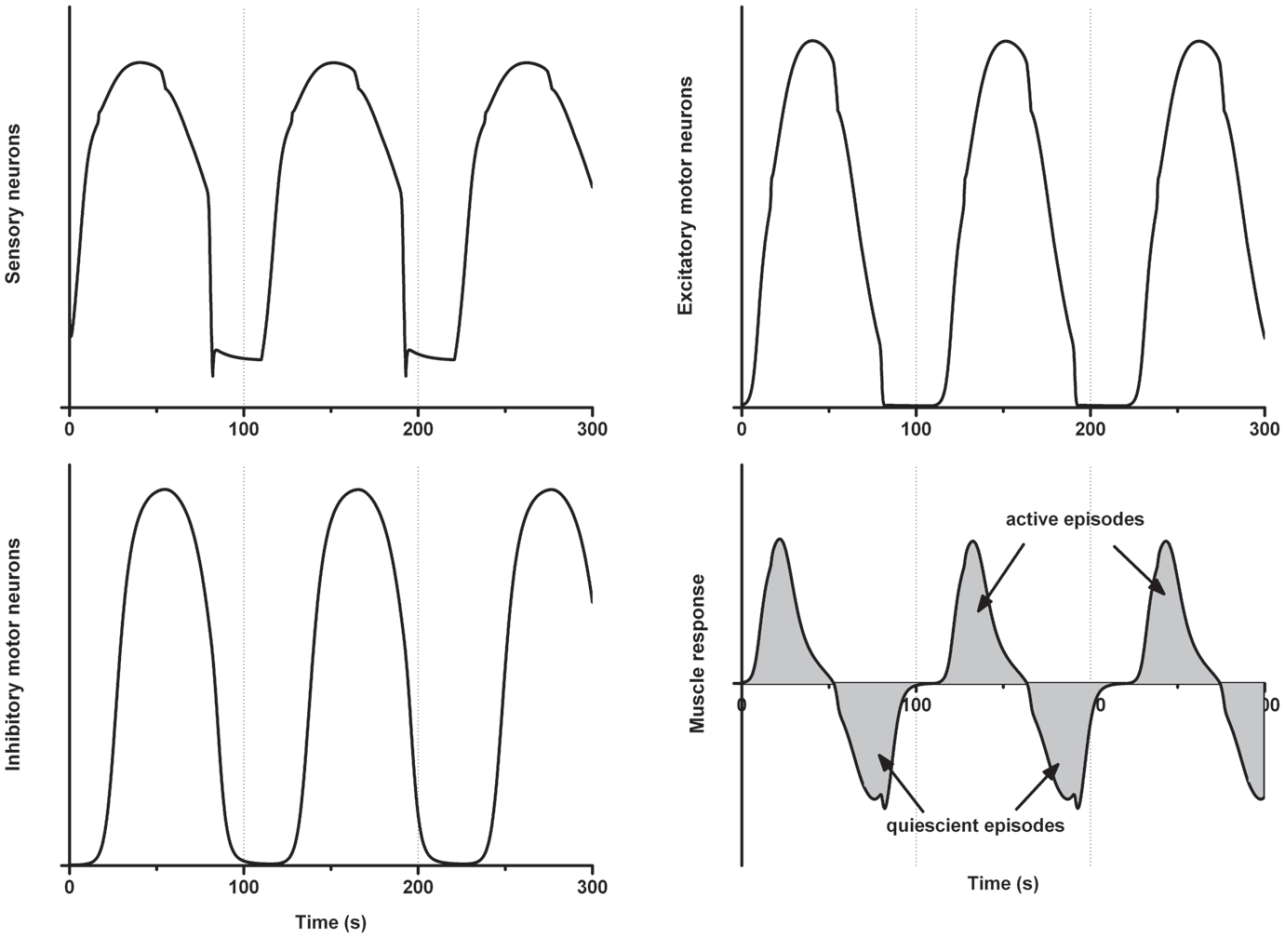
Control output from the computational model. The plots show activity (in arbitrary units) for the different populations of neurons and circular muscle.

The control response model reproduced the oscillation between active and quiescent episodes with the appropriate time courses (Figure 5). The model works as follows. ISNs receive constant input in the form of proximal process potentials (PPPs), which are the membrane potential changes produced in the cell bodies of the ISNs by action potentials conducted antidromically in their axons. In this case, the PPPs represent a nutrient stimulus and create a basal level of drive into ISNs. Activity in ISNs is transmitted to excitatory motor neurons predominantly via fast EPSPs and to inhibitory motor neurons predominantly via slow EPSPs. Due to the different time courses of fast and slow EPSPs, activity in the excitatory motor neurons increases rapidly while activity in inhibitory motor neurons has a longer latency and slower rise time. Accordingly, the activity in the excitatory motor neurons is initially higher than that of the inhibitory motor neurons, causing contractions in the circular muscle. These contractions cause excitatory feedback, which in turn increases PPP input to ISNs. Together these drive ISNs into high levels of activity. This increasing activity initially continues to drive activity in the excitatory motor neurons and so creating positive feedback.

Eventually the ISNs reach their maximum sustainable firing rate, so there is no further increase in the drive onto the excitatory motor neurons. This allows activity in the inhibitory motor neurons, driven by slow EPSPs, to catch up to activity in the excitatory motor neurons. As the activity in the inhibitory motor neurons approaches the level of activity in the excitatory motor neurons, there is less activity in the circular muscle and, hence, less excitatory feedback. The reduction in feedback means there is less input in the form of PPPs to the ISNs so their activity starts to decrease.

The decrease in ISN activity leads to decreased input to both the excitatory motor and inhibitory motor neurons. Transmission to the excitatory motor neurons is via fast EPSPs, so their activity rapidly decreases with the reduced input. However, transmission to inhibitory motor neurons is via slow EPSPs, so while this input to these neurons rapidly decreases, their activity remains high for some time due to the long duration of the slow EPSPs. Thus, activity in the inhibitory motor neurons starts to exceed activity in the excitatory motor neurons. As a result there is no activity in the circular muscle and, hence, no excitatory feedback. The absence of feedback causes the activity in the ISNs to drop, further reinforcing the decline in overall activity.

Eventually activity in the ISNs drops to the basal level determined by the nutrient stimulus in the lumen. With rapid transmission from ISNs to excitatory motor neurons, activity in the latter also quickly reaches its basal level. Activity in the inhibitory motor neurons takes longer to return to basal level due to the duration of the slow EPSPs in these neurons, but does so eventually. Parameters in the model were chosen so that once the ISNs and motor neurons reach low basal firing rates, activity in the excitatory motor neurons is higher than activity in the inhibitory motor neurons. Under these conditions, the network activity starts to build up again, thus initiating another cycle.

#### Varying the network structure

**Figure 6:**
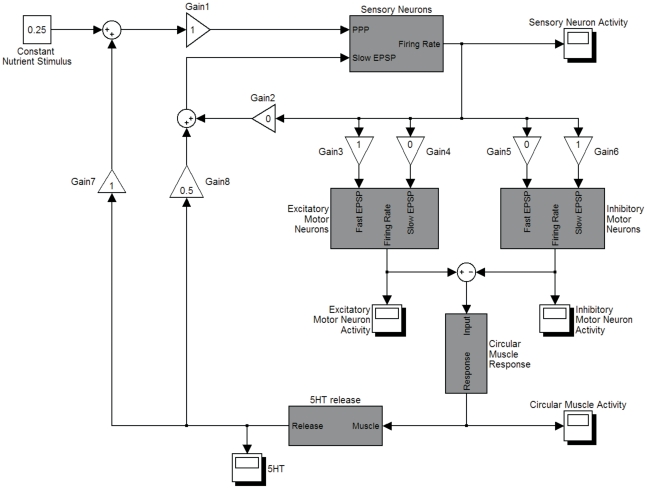
Top level of the Simulink implementation of the model. Lines indicate the flow of signals from one component to the next. The function blocks labelled Sensory Neurons, Excitatory Motor Neurons, Inhibitory Motor Neurons, Circular Muscle Response and serotonin Release contain the appropriate equations described in the methods section, and the implementation of these blocks is in the supplemental material. The Gain blocks determine the strength of that synaptic connection or the strength of the feedback mechanism. All blocks with Activity in the name gather and graph output.

In all the models above and below, there was transmission from ISNs to excitatory and inhibitory motor neurons (Figure 6). Anatomically there are more synapses than this in the ENS. The effects of adding ascending interneurons and descending interneurons were investigated in the model. Both received input from ISNs, but ascending interneurons transmitted activity exclusively to excitatory motor neurons and descending interneurons transmitted activity exclusively to inhibitory motor neurons, forming two separate feedforward pathways. Since the control response required predominantly fast EPSP transmission to excitatory neurons and predominantly slow EPSP transmission to inhibitory neurons, we tested whether these different types of transmission could be located at different points along the feedforward pathways.

In the first scenario, transmission from ISNs to ascending interneurons was predominantly via fast EPSPs and transmission to descending interneurons was predominantly via slow EPSPs. Transmission from both populations of interneurons to their respective motor neurons was predominantly via fast EPSPs. Under these conditions, the durations of the active episodes and quiescent episodes similar to the control behaviours.

In the second scenario, transmission from ISNs to both interneuron populations was predominantly via fast EPSPs. Transmission from ascending interneurons to excitatory motor neurons was predominantly via fast EPSPs, whereas transmission from descending interneurons to inhibitory motor neurons was predominantly via slow EPSPs. Under these conditions the durations of the active episodes and quiescent episodes were also similar to the controls.

Therefore, the model reproduced the same activity patterns for both poly- and monosynaptic transmission to motor neurons. Similar observations were made for all parameter combinations (see below). We will refer to the model without interneurons to simplify the descriptions and discussions, but the same qualitative results can be achieved with interneurons present.

#### Varying the AHP in intrinsic sensory neurons varies the duration of the active episodes

**Figure 7:**
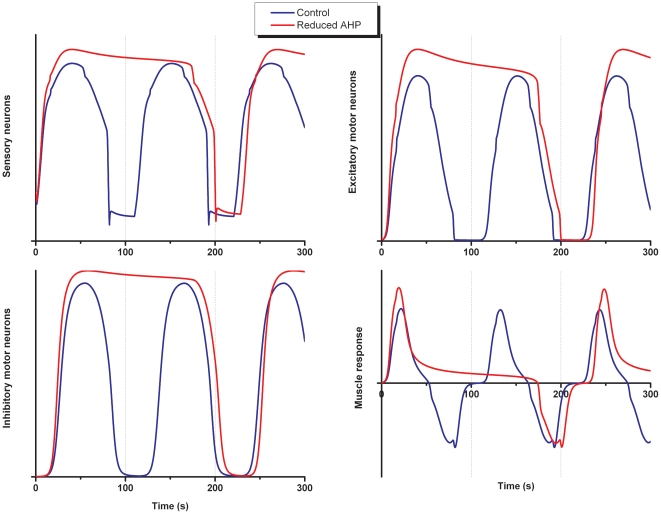
The effect of reducing the size of the AHP. Plots of activity levels (in arbitrary units) for the different populations of neurons and circular muscle for the control response (solid line) and when the size of the AHP in sensory neurons is reduced (K = 0.83 in Eq 7, dotted line).

We mimicked the effect of partially blocking the AHP by reducing *K* in Eq 7. Depressing the AHP, once segmentation has started, resulted in an increase in the duration of active episodes (control: 50 s; reduced AHP: 225 s) without changing the duration of the quiescent episodes. Figure 7 shows activity in the different parts of the system when the size of the AHP is reduced by approximately 20%.

Reducing the size of the AHP increases activity in the ISNs [26], [27]. This is rapidly translated into increased activity in excitatory motor neurons, which in turn is rapidly transmitted to the muscle. Increased muscle activity in turn feeds back onto ISNs. This higher feedback onto ISNs in combination with the reduced internal inhibition (due to the reduced AHP) causes a higher activity level in ISNs and excitatory motor neurons. There is also increased drive onto inhibitory motor neurons, but summation in the second messenger mediated slow EPSP is less than linear in that the maximum depolarization saturates for relatively small inputs and the duration increases only logarithmically with an increasing stimulus [26]. On the other hand, summation of fast EPSPs in the excitatory motor neurons is close to linear in the range around threshold for generation of action potentials. The slow EPSP in the inhibitory motor neurons was close to its maximum during the control response, thus the increased drive from the ISNs did not affect the maximum response of the slow EPSP in the inhibitory motor neurons. Because of the modest increase in the duration of the slow EPSP in the inhibitory motor neurons, activity in this pathway will exceed the activity in the excitatory motor neurons resulting in a similar duration of the quiescent period to control (control: 57 s; reduced AHP: 54 s).

In addition to reducing the size of the AHP, reducing the size of the residual AHP in the presence of a slow EPSP (ρ in Eqs 8 and 9), a well established phenomenon in these neurons [26], [33] was also investigated. This produced very similar results to decreasing the size of the AHP because it effectively decreases the size of the AHP in the presence of a slow EPSP.

**Figure 8:**
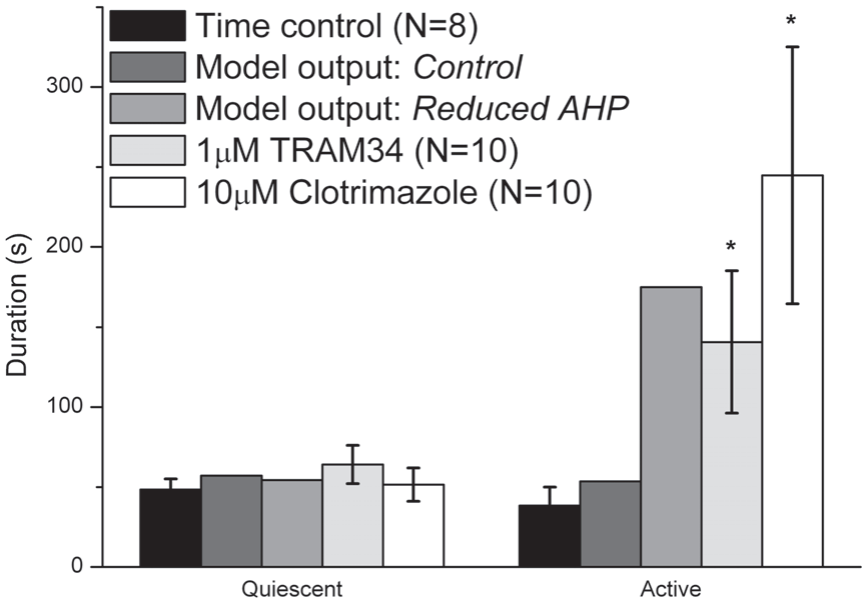
Comparison of model and experiment. Histogram of the duration of the active and quiescent episodes predicted by the computational model and from the *in vitro* pharmacological experiments. There are no error bars on the values predicted by the computational model because there is no variance in these predictions for a given set of parameters. The asterisks indicate significant differences between the control and drug recordings (determined by unpaired t-tests, p<0.05).

In summary, the model reproduces the increase in the duration of active episodes when the size of the AHP is reduced with only a minor affect on the duration of quiescent episodes as seen *in vitro* (Figure 8).

#### Varying synaptic transmission from ISNs to motor neurons

We investigated varying the relative contributions of fast and slow synaptic transmission from ISNs to excitatory or inhibitory motor neurons.

#### Varying fast EPSP transmission to excitatory motor neurons

**Figure 9:**
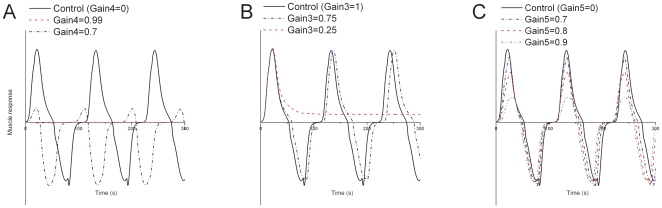
The effect of varying transmission from ISNs to motor neurons. Plots of relative muscle response as the parameters controlling the strength of transmission to motor neurons are varied (Figure 6). *A)* Fast EPSP transmission to excitatory motor neurons. *B)* Slow EPSP transmission to excitatory motor neurons. *C)* Fast EPSP transmission to inhibitory motor neurons.

Decreasing the fast EPSP transmission from ISNs to excitatory motor neurons resulted in less drive to the circular muscle, less excitatory feedback, and a shorter duration of the active episodes (Figure 9A). If the fast EPSP transmission is decreased enough, there is no activation of excitatory motor neurons and thus no activity in the circular muscle. As transmission from ISNs to inhibitory motor neurons was unchanged, the duration of the quiescent episodes showed only minor variations.

As fast transmission to excitatory motor neurons is mediated by nicotinic acetylcholine receptors [34], [35], [36] this predicts that sub-maximal concentrations of nicotinic antagonists will decrease the duration of active episodes without altering the duration of quiescent episodes.

#### Varying slow EPSP transmission to excitatory motor neurons

The strength of slow EPSP transmission from ISNs to excitatory motor neurons was increased, while other properties of these slow EPSPs were the same as those in inhibitory motor neurons. Large increases in the strength of slow EPSP transmission from ISNs to excitatory motor neurons had very little affect on the duration of the active and quiescent episodes (Figure 9B), as long as the strength of the slow EPSPs in the excitatory motor neurons was less than that of the slow EPSPs in the inhibitory motor neurons. Under these conditions, the level of activity in the inhibitory motor neurons could still easily approach the level of activity in the excitatory motor neurons to turn off the excitatory feedback loop. If the strength of slow EPSP transmission from ISNs to excitatory neurons was almost equal to, or greater than, that of ISNs to inhibitory motor neurons, oscillations were not possible. This is because activity in inhibitory motor neurons cannot overtake activity in excitatory motor neurons, which leaves the circular muscle in a constant state of contraction, preventing oscillations.

This predicts that slow EPSPs in excitatory motor neurons are smaller than those in inhibitory motor neurons for the same input.

#### Varying fast EPSP transmission to inhibitory motor neurons

Increasing the strength of fast EPSP transmission from ISNs to inhibitory motor neurons had little affect when the strength was below that of fast EPSP transmission from ISNs to excitatory neurons (Figure 9C). However, as the strength of the fast EPSP transmission from ISNs to inhibitory motor neurons approached that of ISNs to excitatory motor neurons a small decrease in the durations of the active episodes (54 s to 47 s for Gain5 = 0.9 in Figure 6) and the quiescent episodes was observed (57 s to 49 s for Gain5 = 0.9 in Figure 6). This was because of the smaller difference in activity between the excitatory motor neurons and inhibitory motor neurons so activity in the latter caught up faster. Since activity in the inhibitory motor neurons caught up faster, all neurons were at lower firing rate when the excitatory feedback was switched off. This lower firing rate resulted in less slow EPSP activity in the inhibitory motor neurons, which caused the reduced duration of the quiescent episodes. If the strength of fast EPSP transmission from ISNs to inhibitory neurons was almost equal to, or greater than, that of ISNs to excitatory motor neurons, oscillations were no longer possible. This is because activity in the inhibitory motor neurons closely follows activity in the excitatory motor neurons, which cancels out at the level of circular muscle. Thus, the build up of activity in response to the basal firing rate in ISNs, as in the control response, is prevented.

This predicts that fast EPSP transmission to inhibitory motor neurons is weaker than fast transmission to excitatory motor neurons.

#### Varying slow EPSP transmission to inhibitory motor neurons

Decreasing the strength of slow EPSP transmission from ISNs to inhibitory motor neurons (Gain6 in Figure 6) increases the duration of active episodes. This is because it takes longer for the inhibitory drive from these neurons to overtake excitatory drive to the muscle from excitatory motor neurons. The decreased slow input into the inhibitory motor neurons also means that the slow EPSPs do not last as long once drive from the ISNs decreases, which causes a decrease in the duration of the quiescent episodes. With further decreases in the strength of the slow EPSP transmission, activity in the inhibitory motor neurons can no longer overtake drive from the excitatory motor neurons and oscillations are no longer possible. In this case, the circular muscle reaches a stable steady state of constriction.

This predicts that sub-maximal concentrations of antagonists that prevent slow EPSP transmission from ISNs to inhibitory motor neurons will decrease the duration of quiescent episodes and increase the duration of active periods. Furthermore, this predicts that complete blockade of slow EPSP transmission from ISNs to inhibitory motor neurons will abolish quiescent episodes and leave the circular muscle in a constant state of activity.

#### Varying slow EPSP transmission between ISNs

Positive synaptic feedback within the ISN population and its interaction with AHPs seen in these neurons is necessary for sensory transduction [26], [27]. In the control model, there was no slow EPSP transmission between ISNs. Including slow EPSP transmission between ISNs (Gain2 in Figure 6) increased the duration of the active and quiescent episodes. Increased activity in the ISNs meant there was more drive onto excitatory motor neurons and, therefore, it took longer for the activity in the inhibitory motor neurons to build up and turn off the positive feedback. The increased drive into inhibitory motor neurons increased the duration of synaptic events in these neurons causing longer duration inhibitory input to the muscle.

If the strength of slow EPSP transmission between ISNs was strong enough, then activity in the ISNs remained at a high stable firing rate regardless of activity in other parts of the system and oscillations were not possible.

#### Varying sensory neuron feedback from the circular muscle

Many ISNs are tension sensitive [37] and so will be excited when the muscle actively contracts (see [15] for full discussion). Furthermore, muscle contraction is known to release serotonin from the mucosa, which will cause both PPPs and slow EPSPs in the ISNs [20], [38]. With no feedback from the muscle (setting Gain7 = 0 and Gain8 = 0, Figure 6) oscillations between quiescent and active episodes were not possible.

## Discussion

After a meal, the intestine engages a highly complex motor program to mix food with secretions and bring the products of digestion into contact with the absorptive epithelium. This motor program consists of several different types of contractions, either stationary contractions, contractions that propagate slowly over short distances or rapidly propagating, long distance contractions [10]. In the guinea-pig jejunum, these motor patterns are generated by local circuits within the ENS since they occur in isolated preparations *in vitro* and are blocked by agents that block action potential generation and fast excitatory synaptic transmission.

### Two independent pacemakers

Two distinct repetitive patterns were identified in the presence of luminal decanoic acid. The first was the alternation of activity and quiescence whose properties were clearly modified by blockade of I_K_ potassium channels, which we have called the *driving* pattern. The second consisted of much higher frequency bursts of stationary and SL contractions that occurred at constant locations within the jejunal segment, which we termed *isolated bursts*. The isolated bursts are significant components of active contraction episodes, but less organized contractile activity is also observed. The widely disparate frequencies of the two patterns suggest that they arise from different mechanisms with the driving mechanism alternately activating or inhibiting the isolated burst mechanism. The effects of inhibiting I_K_ channels support this conclusion with the duration of active contraction periods and the incidence of isolated bursts increasing, but with other properties of isolated bursts being unaffected. Thus, the pacemaker responsible for the repeated contractions within the isolated bursts is independent of I_K_ channels, in direct contrast to the slower driving pacemaker responsible for setting the activity of the whole system.

Immunohistochemisty has shown that I_K_ channels are expressed on enteric neurons in the mouse [39], rat [40] and humans [41]. In rat, I_K_ channels appear confined to Dogiel type II neurons that are immunoreactive for calretinin, a marker for ISNs in this species. Also, I_K_ channel block had no effect on 19 out of 19 non-sensory neurons [42]. Furthermore, the application of the TRAM-34 and clotrimazole (at the concentrations used in this present study) decreases the AHPs in ISNs to approximately 15% of control in guinea-pig [31]. I_K_ channels may also be present in the epithelial cells of guinea-pig jejunum because they have been identified in rat colonic epithelial cells [43]. Activation of I_K_ channels in enterocytes stimulates fluid secretion [44], and this could have an indirect effect on segmentation. Further, clotrimazole-sensitive potassium currents have been seen in ICC in mice [45]. Even though there is little slow-wave activity in the guinea-pig, if the I_K_ channels blockers were acting on ICCs, they would be expected to influence the properties of isolated bursts, rather than the contractile episodes, because of the time course of potassium currents in these cells [46]. However, I_K_ currents in ISNs have a much longer time course, which would be expected to influence the properties of contractile episodes rather than isolated bursts. Given that the ENS is active during segmentation [10] and suppressing the AHP increases activity in ISNs [26], it is no surprise that blocking I_K_ channel produces more contractile activity [25]. Therefore, it appears that the I_K_ channels on ISNs are the most likely site of action in this preparation. Furthermore, blocking I_K_ channels increased the duration of active episodes, but not quiescent episodes. Any explanation or model must account for this difference.

Neither isolated bursts nor the driving pacemaker depend on 5-HT_1A_ receptor activation, because they are unaffected by NAN-190 and WAY-100135 at concentrations known to block 5-HT_1A_ receptors within the ENS [28], [47]. Limited studies of inhibitory synaptic potentials (IPSPs) in myenteric neurons indicate that these might be mediated by 5-HT_1A_ receptors [28]. It has been found that 5-HT_1A_ receptors are involved in pre-synaptic inhibition of transmitter release [48], [49]. While it is possible that blocking IPSPs in ISNs and blocking inhibition of transmitter release could cancel each other out, blocking inhibition of transmitter release involved in transmission to excitatory motor neuron would make these two actions synergistic. However, the current results suggest that 5-HT_1A_ receptors are not involved in either the high frequency pattern generator or in the low frequency driving pattern generator. NAN-190 also blocks α_2_-adrenoceptors [50]. However, if this occurred in our experiments, NAN-190 and WAY-100135 would produce different results, but as each had no effect it is unlikely α_2_adrenoceptors influence segmenting motor patterns in the absence of sympathetic stimulation *in vitro*.

Blocking I_K_ channels has no effect on WL contractions, but increases SL and stationary contractions. The same I_K_ channel blockers can cause a switch from propulsive motor patterns to mixing motor patterns in un-fed rats *in vivo* [25] and it has been suggested that this is a result of preventing coordinated firing in populations of ISNs [26]. However, in the current study, where the mixing motor pattern was induced with decanoic acid in the lumen to mimic the fed-state, the AHPs in ISNs apparently have no influence on propulsive contractions. This indicates that there are separate mechanisms controlling segmenting and propulsive contractions in the fed-state, although both are dependent on the driving pattern generator. The idea that the propulsive contractions are regulated by a separate rhythmic pattern generator from the one that sets the frequency within segmentation bursts is consistent with recent findings on the effects of acute cholera toxin treatment on motility in this same preparation [30].

### Model interpretation

Our abstract model allowed exploration, at a qualitative level, of how the many nonlinear elements involved in segmentation might interact to produce the oscillation between active and quiescent episodes. Construction of the model followed established methodology [51], in which physical quantities such as membrane potential, internal calcium concentration, firing rate and amount of phosphorylated channel, etc are averaged over a homogenous population of neurons. The model describes activity in populations of ISNs, inhibitory and excitatory motor neurons and the circular smooth muscle. Signals within and between these components included sensory input from nutrient in the mucosa, fast EPSPs, slow EPSPs, AHPs and mechanically driven excitatory feedback from the muscle. Episodes of contractile activity occur when activity in the excitatory motor neurons exceeded activity in the inhibitory motor neurons.

#### Segmentation requires muscle feedback

Parameters controlling the time course or duration of events are available from physiological data and were incorporated into the model. Specifically, these include the dynamics and nonlinear summation of slow EPSPs [52] and the dynamics of feedback to ISNs from contractions in the circular muscle [20]. Cycling between active and quiescent episodes was only possible with feedback from the circular muscle. Furthermore, to reproduce the time scale of these slow oscillations, around 120 s for the complete cycle, there needs to be a component in the physical system with a response time of tens of seconds. Candidates for this are tachykinin mediated sEPSPs in ISNs [28], [53] and contraction mediated mucosal serotonin release [20]. The former is unlikely because NK_3_ receptor antagonists have no effect on segmentation (Gwynne and Bornstein, unpublished observations). Thus, the model makes the novel prediction that contraction induced serotonin release plays an important role in the fed-state motor pattern.

Recently, there has been some conjecture as to whether serotonin release from the mucosa is required for intestinal motor patterns. For example, colonic migrating motor complexes have been reported to be abolished by removing the mucosa by some authors [54] but not affected by others [55]. However, studies using the *in vitro* segmentation preparation suggest infusing the lumen with decanoic acid activates the ENS via serotonin release from the mucosa [56]. Therefore, even if serotonin release from the mucosa is not required for some motor patterns, its presence dramatically influences other motor patterns observed *in vitro*.

Serotonin from the mucosa activates 5-HT_3_ receptors on ISN terminals resulting in PPP and action potential discharge. Furthermore, serotonin applied to the mucosa elicits slow EPSPs in ISNs [17], [57]. The model produces active and quiescent episodes when there was either PPP feedback and/or slow EPSP feedback. Without feedback from the circular muscle, the model will not cycle between active and quiescent episodes. This suggests an alternative to the usual idea that serotonin released from the mucosa initiates a single peristaltic contraction. In our model, serotonin released from the mucosa increases the excitability of ISNs, making it more likely that bursts of stationary contractions in the circular muscle will be observed.

#### Transmission is fast in the excitatory pathway and slow in the inhibitory pathway

The model includes transmission from ISNs to both excitatory and inhibitory motor neurons. This may be via mono- or poly-synaptic pathways. The model predicts that transmission from ISNs to excitatory motor neurons is rapid, and that there is a delay in transmission to inhibitory motor neurons. Thus transmission to excitatory motor neurons is predominantly by fast EPSPs and by slow EPSPs to inhibitory motor neurons. Furthermore, the model was robust enough to allow both types of transmission to both types of motor neurons, provided fast EPSP transmission to excitatory motor neurons was greater than fast EPSP transmission to inhibitory motor neurons and slow EPSP transmission to excitatory motor neurons was less than slow EPSP transmission to inhibitory motor neurons. Without these differences in synaptic transmission, the cycling between active and quiescent episodes did not occur. The neurotransmitters and receptors involved in transmission from ISNs directly to excitatory motor neurons have not been identified, but pharmacological studies suggest that transmission via ascending interneurons involves fast nicotinic receptors and slow neurokinin 3 (NK_3_) receptors [34], [36]. Thus, the model is consistent with the literature that transmission from sensory neurons to excitatory motor neurons involves both fast and slow EPSP transmission.

Our model predicts agents that partially block fast EPSPs in excitatory motor neurons will decrease active episode durations or, if strong enough, abolish them. Indeed, hexamethonium abolishes segmentation [9], which might be due to blocking fast EPSPs in excitatory motor neurons.

Slow EPSPs can be evoked in inhibitory motor neurons via both direct and indirect pathways from ISNs [58], [59]. The model predicts that increasing slow EPSP transmission to inhibitory motor neurons will increase the duration of quiescent episodes and decrease the duration of active episodes, and decreasing the same slow EPSP transmission has the opposing effects. Furthermore, the model predicts that increasing activity in ISNs will increase overall activity, but the duration of the quiescent episodes will not be altered. This prediction has recently been confirmed by a study showing no change in quiescent episodes, but an increase in the contraction frequency and duration of episodes of propulsive contractions after short-term exposure to cholera toxin [30].

### Conclusion

In summary, this study has shown that the duration of the active contractile periods during segmentation in the guinea-pig jejunum *in vitro* depends on a complex interplay between AHPs in ISNs, fast and slow EPSPs in excitatory and inhibitory pathways (respectively) and on feedback from the contracting muscle, possibly via release of serotonin from the mucosa. In species exhibiting strong slow waves, such as human, the properties of contractions during active contractile periods would be determined by the interaction of the high frequency neural pattern generator and slow waves However, the low frequency oscillation between active and quiescent contractile periods is likely to be neurally mediated in these species as well. This work predicts that blocking feedback from contracting muscle will abolish contractile periods during segmentation and that synaptic inputs to excitatory pathways differ qualitatively to those in inhibitory motor pathways.

## Materials and Methods

### Ethics Statement

*In vitro* experiments were conducted in accordance with the guidelines of the National Health and Medical Health and Medical Research Council of Australian and with approval from the University of Melbourne Animal Experimentation Ethics Committee (Approval number 0808369.2).

### *In vitro* experiments

The *in vitro* model of segmentation has been previously published [10] and is only summarised here. Guinea-pigs (250–400 g) of either sex were killed by being stunned and then having their spinal cords severed. Segments of mid-jejunum, 6–7 cm long, were dissected free and placed in a horizontal organ bath. Physiological saline (composition, mM: NaCl 118, KCl 4.6, CaCl2 2.5, MgSO4 1.2, NaH2PO4 1, NaHCO3 25, D-Glucose 11, bubbled with 95% O2 and 5% CO2) was flushed through the lumen to clear any contents. The tissue was placed in the organ bath and physiological saline was superfused into the bath at a flow rate of approximately 6 ml per minute, bath temperature was 37°C. Cannulae were inserted into both ends of the intestinal segments and tied down with thread. The oral cannula was attached to a reservoir of physiological saline, allowing the contents to be flushed through the lumen. The anal cannula was attached to a vertical outflow tube.

Once the preparation was set up, it was left to equilibrate for 30–60 minutes. During this period, a 20 minute video recording of spontaneous activity was made and the pressure threshold for initiation of peristaltic contractions was determined by raising the inflow pressure in steps of 1 cmH_2_O at intervals of approximately 30 seconds. The lumen was then flushed with saline solution containing decanoic acid (Sigma Aldrich, NSW, Australia) at a concentration of 1 mM. Over the following 120–180 minutes, video recordings were made in 20 minute blocks. Time to onset of segmentation varies between preparations [10] and is defined as the first display of stationary or short-length propagating contractions. A 20 minute control recording was made at the onset of segmentation. Drugs were added to the physiological saline superfused into the bath and allowed to wash in for 20 minutes. After wash in, another 20 minute recording was made. Finally, the drug was removed from the inflow and replaced with Krebs again. 2–4 20 minute recordings were then made as the drug washed out.

Video recordings were processed using in house software that measures the intestinal diameter for each frame of the video recording [10], [60]. The software produces spatiotemporal maps which are two dimensional plots with intestinal length on the X-axis, time on the Y-axis and intestinal diameter represented by a grey scale. Contractions were identified by eye and classified as whole length (WL) contractions, short length (SL) contractions or stationary contractions, according to published criteria [10]. Briefly, WL contractions appeared at either the oral or the anal end of the preparation and propagated to the other end. SL contractions were initiated at various different locations of the preparation and propagated slowly either orally or anally, with the amplitude decreasing over 1–2 cm, so that they disappeared before reaching the end of the segment. For each contraction, its propagation speed and length were measured.

The following criteria were used to organize the individual contractions into larger patterns. A period of quiescence was defined as 20 seconds or longer without a contraction. This period of time was chosen as a balance between unrealistically short quiescent episodes and the maximum quiescent episode observed (40 s). An active period was defined as any time that was not a quiescent episode. The durations of active and quiescent episodes were measured.

An *isolated burst of contractions* was defined as three or more contractions of a similar length (within 50% of each contraction length), occurring at similar intervals (within 50%), at similar locations (the centre of each contraction was within the most oral and most anal edges of the largest contraction within the burst) and with no other contractions passing through the same region during the isolated burst. The rate at which isolated bursts were observed and the properties of each burst were also determined. The measured properties of each burst included the number of contractions in a burst and the frequency of contractions in a burst.

Data are reported as mean ± standard error of the means. Statistical comparisons of the mean values were made using unpaired t-tests, with P values <0.05 taken as significant. No correction for multiple comparisons were made.

### Pharmacological agents

TRAM34 (1-[(2-chlorophenyl)dipenylmethyl]-1H-pyrazole), clotrimazole, NAN-190 (Sigma Aldrich, NSW, Australia) and WAY-100135 (Tocris Cookson, Bristol, UK) were applied to the serosa through the bath perfusate. TRAM34 was mixed with pure dimethyl sulfoxide (DMSO) with the assistance of a vortex mixer at room temperature to create a stock solution of 1 mM. Clotrimazole was also mixed with pure DMSO to create a stock solution of 10 mM. NAN-190 was mixed with 10% DMSO in distilled water alternated between a vortex mixer and sonic machine for 2 hours until completely dissolved to create a stock solution of 1 mM. WAY-100135 was mixed with distilled water to create a stock solution of 1 mM. All stock solutions were stored at −20°C. Working dilution solutions of TRAM34 were made up on the day by slowly mixing the stock solution with physiological saline that had been gassed with 95% O_2_-5% CO_2_ for at least 30 minutes [31].

### The computer model

**Figure 10:**
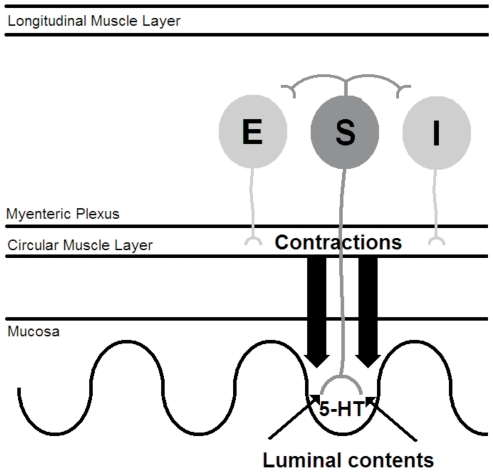
A cartoon of the physiological feedback model. The luminal contents stimulate the ISNs which in turn activate excitatory motor neurons and inhibitory motor neurons. When activity in the excitatory motor neurons exceeds activity in the inhibitory motor neurons, there are contractions in the circular muscle. These contractions cause the release of serotonin from the mucosa which further stimulates the ISNs.

We used a lumped or population model [51] to describe activity in a length of gut small enough that the activity in the population can be considered homogeneous. We used state variables to describe mean firing rate of ISNs, concentrations of several intracellular messengers, inhibitory motor neurons, excitatory motor neurons and the mechanical state of the circular muscle (Figure 10). Signals consisted of fast excitatory post synaptic potentials (EPSPs), slow EPSPs, proximal process potentials (PPPs) and AHPs. The model was implemented in Simulink (Mathworks, USA). The top level of the model is shown in Figure 6 and the detailed model is presented in the Supplementary material. The model is available from ModelDB (http://senselab.med.yale.edu/modeldb/) or by contacting the authors.

Application of chemicals to the mucosa causes action potentials in ISNs that do not arise from synaptic activity [17], [57], [61]. In this model, to represent a nutrient stimulus (such as decanoic acid in the lumen), ISNs received a constant input in the form of PPPs, which rapidly increase the activity in these neurons. Output from ISNs to excitatory and inhibitory motor neurons was tested using all combinations of fast and/or slow EPSPs. Activity in the excitatory motor neurons then excites the circular muscle and activity in the inhibitory motor neurons inhibits activity in the circular muscle. Activity in the circular muscle causes the release of serotonin [20], [55], [62] which, in turn, activates ISNs via PPPs and/or slow EPSPs [17], thereby creating the feedback loop. Note, the implementation of this feedback does not depend on the underlying mechanism being release of serotonin, so the model's predictions are not dependent on serotonin release *per se*.

#### Detailed Description

*Motor neuron population model.* In the model, motor neurons can receive both fast and slow EPSP input. The contribution to the average membrane potential is determined separately for each type of synaptic input and these are then combined to determine population firing rate.

The equation to describe the response to fast EPSP input is a simple sigmoid function because a fast EPSP has very little transmission delay and a short time course compared to other time scales in the model. This is a typical response function for models of this sort [51], [63]. The equation is:
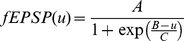
(1)where u is the rate of presynaptic action potentials and the constants A, B and C are chosen to provide a physiologically plausible response.

Responses to slow EPSP have long latencies, long durations and sum nonlinearly. These properties are reproduced by the following model [52]:
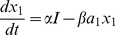
(2)

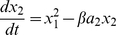
(3)

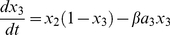
(4)where *I* is the presynaptic firing rate, *x_1_* is the amount of diffusible second messenger, *x_2_* is the amount of second stage reactant or catalytic subunit, *x_3_* is the amount of phosphorylated potassium channel, *a_1_*, *a_2_* and *a_3_* are constants that determine the shape the slow EPSP and have been derived from experimental measurements of responses in S neurons [52]. The additional parameters *α* and *β* are not required mathematically but are a useful parameterization for exploring the consequences of varying the relative strength of the slow EPSP input and the rate of decay for the slow EPSP current.

We used a sigmoid like function to convert the average amount of phosphorylated channel into an average membrane potential depolarization, again as is usual for lumped models [51], [63]. The function to convert the average amount of phosphorylated channel to average membrane potential depolarization is
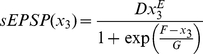
(5)where *x_3_* is the output from Eq 4, *D* is a constant that determines the maximum firing rate, *E* and *G* determine the rate of rise of action potential firing rate after the threshold is reached and *F* determines the threshold for the rapid increase in action potential firing rate. This function has a shallow initial curve, which has the effect of reducing the action potential firing rate to close to zero until a threshold is reached, then the firing rate rapidly increases to a high level. The firing rate function has this form for two reasons. Firstly, in a single neuron when the slow EPSP amplitude is increased above action potential threshold it will continue to increase the firing rate. Secondly, for a population of neurons, as the slow EPSP input is increased, the resulting slow EPSP current will drive more neurons to action potential threshold, causing a higher firing rate in the population of neurons.

The firing rate of the S neuron population is determined from the combination of fast and slow synaptic inputs. In a single neuron, the absolute and relative refractory periods prevent action potentials from firing at arbitrarily high firing rates so the total firing rate will not be a simple linear combination of the fast and slow EPSP activity. Furthermore, previous modelling of S neuron populations indicates the combined firing rate is only a small amount higher than the highest individual firing rate [64]. Therefore, the equation used to describe the total firing rate is:
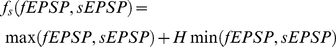
(6)where *fEPSP* is the output from Eq 1 and *sEPSP* is the output from Eq 5, *H* is a constant that determines the proportion of the lower individual firing rate that contributes to the total firing rate.

### The ISN network model

A lumped model was used to define the overall activity within a population of ISNs. These neurons have two types of inputs, PPPs and slow EPSPs, and have a prominent calcium mediated AHP. Calcium opens potassium channels that the slow EPSP is closing [33] and this interaction is likely to be important in controlling firing in these neurons [26]. Calcium entering during the action potentials provide negative feedback onto the network firing rate.

The population average internal calcium concentration is given by
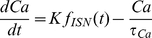
(7)where f_ISN_ is the ISN firing rate and τ_Ca_ is the time constant of decay for the calcium transient. As with motor neurons, the amount of phosphorylated channel in ISNs is determined by Eqs 2–4, but with different parameters to reproduce the different time course of slow EPSPs in these neurons. The overall effect of the slow EPSP and internal calcium on the population average membrane potential is [26]

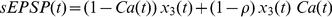
(8)where ρ determines the size of the residual AHP in the presence of a slow EPSP. The residual Ca mediated effect on the population average membrane potential is
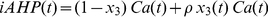
(9)The effect of PPP input on the population averaged membrane potential is described by a sigmoid function similar to Eq 1. This is used because a PPP also has small delay and a short time course. Also, PPPs readily evoke action potentials, provided the input is strong enough. Any Ca mediated current suppresses firing driven by PPP input. As with S neurons the total firing rate is given by
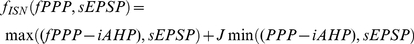
(10)This abstract model for sensory neuron populations contains previously used mathematical models for single neurons to estimate the slow EPSP conductance, AHP activity, and the interaction between the slow EPSP and AHP. However, it is combined with lumped models for the generation of action potential activity from PPPs and slow EPSPs. This combination is able to reproduce previously published responses in ISN networks for different input frequencies and varying the residual AHP in the presence of slow EPSPs [64].

### A model of circular muscle contraction

The response of the circular muscle to inhibitory and excitatory motor neuron input was calculated as
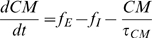
(11)where f_E|I_ is the firing rate of the excitatory or inhibitory motor neuron population, CM is the state of the circular muscle and τ_CM_ is the muscle time constant.

### Serotonin in the lumen

Serotonin is released from the mucosa when contractions occur in the circular muscle [20]. The release of serotonin occurs within a few hundred milliseconds of the circular muscle contraction and the serotonin concentration returns to baseline within 30 s. To model this response it was assumed the serotonin release occurred instantaneously, had a long decay and was directly proportional to the amount of activity in the circular muscle. The equation for this response was:
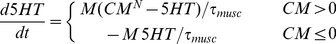
(12)where *M* is a constant that determines the time course of the serotonin release, *N* is a constant that adjusts the level of activity in the contractile apparatus to determine the threshold and maximum to evoke serotonin release, *τ_musc_* is the serotonin release time constant and *CM* is the activity in the contractile apparatus as given by Eq (11). When the activity in the contractile apparatus was dominated by activity in the inhibitory motor neurons, it was assumed the muscle would be dilated and, therefore, there would be no serotonin release.

## Supporting Information

Table S1 Contraction rates prior to drug application, in the presence of the drug and after drug wash out. p<0.05 are highlighted in bold.
(DOC)

Table S2 Contraction rates for the whole-length propagating (WL) contractions in the presence of the drug.
(DOC)

Table S3 Contraction rates for the short-length propagating (SL) contractions in the presence of the drug. p<0.05 are highlighted in bold.
(DOC)

Table S4 Contraction rates for the stationary contractions in the presence of the drug. p<0.05 are highlighted in bold.
(DOC)

Table S5 This table provides the measurement data for properties of whole-length propagating (WL) contractions and stationary contractions in the presence of the drug. p<0.05 are highlighted in bold.
(DOC)

Table S6 This table provides the measurement data for properties of orally and anally directed short-length propagating (SL) contractions in the presence of the drug. p<0.05 are highlighted in bold.
(DOC)

Table S7 This table provides the data on the effects of drugs on bursts of contractions. p<0.05 are highlighted in bold.
(DOC)

Table S8 Drug effects on the duration of active and quiescent episodes. p<0.05 are highlighted in bold.
(DOC)
