# Supplementary material for: Multiple Neural Oscillators and Muscle Feedback Are Required for the Intestinal Fed State Motor Program
Source: PLoS One. 2011 May 5;6(5):e19597. doi: 10.1371/journal.pone.0019597 (PMC3088688; doi:10.1371/journal.pone.0019597)
Supplement: Table S1 — Contraction rates prior to drug application, in the presence of the drug and after drug wash out. p<0.05 are highlighted in bold. (DOC) [file pone.0019597.s001.doc]

|  | Control | | | Drug | | | Washout | | |
| --- | --- | --- | --- | --- | --- | --- | --- | --- | --- |
|  | min^-1^ | N | P | min^-1^ | N | P | min^-1^ | N | P |
| Control | 4.3 ± 0.7 | 9 |  | 8 ± 2 | 9 |  | 10 ± 3 | 9 |  |
| TRAM34 | 5.7 ± 0.9 | 10 | 0.249 | 17 ± 2 | 10 | **0.022** | 11 ± 2 | 10 | 0.828 |
| Clotrimazole | 3.6 ± 1.1 | 10 | 0.626 | 28 ± 7 | 10 | **0.019** | 37 ± 5 | 10 | **0.001** |
| NAN-190 | 3.3 ± 1.7 | 7 | 0.578 | 14 ± 5 | 7 | 0.339 | 9 ± 2 | 7 | 0.783 |
| WAY-100135 | 3.6 ± 1.0 | 6 | 0.571 | 10 ± 2 | 6 | 0.585 | 13 ± 1 | 6 | 0.423 |
