# Supplementary material for: Multiple Neural Oscillators and Muscle Feedback Are Required for the Intestinal Fed State Motor Program
Source: PLoS One. 2011 May 5;6(5):e19597. doi: 10.1371/journal.pone.0019597 (PMC3088688; doi:10.1371/journal.pone.0019597)
Supplement: Table S2 — Contraction rates for the whole-length propagating (WL) contractions in the presence of the drug. (DOC) [file pone.0019597.s002.doc]

|  | Total WL contractions | | | Anal WL contractions | | | Oral WL contractions | | |
| --- | --- | --- | --- | --- | --- | --- | --- | --- | --- |
|  | min^-1^ | N | P | min^-1^ | N | P | min^-1^ | N | P |
| Control | 0.39 ± 0.03 | 9 |  | 0.4 ± 0.1 | 9 |  | 0.006 ± 0.006 | 9 |  |
| TRAM34 | 0.36 ± 0.07 | 10 | 0.784 | 0.36 ± 0.07 | 10 | 0.786 | 0.005 ± 0.005 | 10 | 0.934 |
| Clotrimazole | 0.19 ± 0.05 | 10 | 0.070 | 0.19 ± 0.05 | 10 | 0.081 | 0 ± 0 | 10 | 0.305 |
| NAN-190 | 0.43 ± 0.10 | 7 | 0.778 | 0.4 ± 0.1 | 7 | 0.823 | 0.01 ± 0.01 | 7 | 0.541 |
| WAY-100135 | 0.20 ± 0.03 | 6 | 0.131 | 0.20 ± 0.03 | 6 | 0.148 | 0 ± 0 | 6 | 0.435 |
