# Supplementary material for: Multiple Neural Oscillators and Muscle Feedback Are Required for the Intestinal Fed State Motor Program
Source: PLoS One. 2011 May 5;6(5):e19597. doi: 10.1371/journal.pone.0019597 (PMC3088688; doi:10.1371/journal.pone.0019597)
Supplement: Table S3 — Contraction rates for the short-length propagating (SL) contractions in the presence of the drug. p<0.05 are highlighted in bold. (DOC) [file pone.0019597.s003.doc]

|  | Total SL contractions | | | Anal SL contractions | | | Oral SL contractions | | |
| --- | --- | --- | --- | --- | --- | --- | --- | --- | --- |
|  | min^-1^ | N | P | min^-1^ | N | P | min^-1^ | N | P |
| Control | 2.3 ± 0.6 | 9 |  | 1.6 ± 0.3 | 9 |  | 0.7 ± 0.3 | 9 |  |
| TRAM34 | 4.3 ± 0.7 | 10 | **0.037** | 3.1 ± 0.5 | 10 | **0.023** | 1.3 ± 0.3 | 10 | 0.174 |
| Clotrimazole | 10 ± 3 | 10 | **0.034** | 7 ± 2 | 10 | **0.032** | 3 ± 1 | 10 | **0.041** |
| NAN-190 | 5 ± 2 | 7 | 0.184 | 3 .1± 1.2 | 7 | 0.195 | 2.0 ± 1.0 | 7 | 0.179 |
| WAY-100135 | 2.2 ± 0.6 | 6 | 0.943 | 1.6 ± 0.4 | 6 | 0.925 | 0.61 ± 0.1 | 6 | 0.749 |
