# Supplementary material for: Multiple Neural Oscillators and Muscle Feedback Are Required for the Intestinal Fed State Motor Program
Source: PLoS One. 2011 May 5;6(5):e19597. doi: 10.1371/journal.pone.0019597 (PMC3088688; doi:10.1371/journal.pone.0019597)
Supplement: Table S4 — Contraction rates for the stationary contractions in the presence of the drug. p<0.05 are highlighted in bold. (DOC) [file pone.0019597.s004.doc]

|  | Total stationary contractions | | |
| --- | --- | --- | --- |
|  | min^-1^ | N | P |
| Control | 6 ± 2 | 9 |  |
| TRAM34 | 12 ± 2 | 10 | **0.025** |
| Clotrimazole | 18 ± 4 | 10 | **0.014** |
| NAN-190 | 8 ± 3 | 7 | 0.504 |
| WAY-100135 | 8 ± 1 | 6 | 0.420 |
