# Supplementary material for: Multiple Neural Oscillators and Muscle Feedback Are Required for the Intestinal Fed State Motor Program
Source: PLoS One. 2011 May 5;6(5):e19597. doi: 10.1371/journal.pone.0019597 (PMC3088688; doi:10.1371/journal.pone.0019597)
Supplement: Table S5 — This table provides the measurement data for properties of whole-length propagating (WL) contractions and stationary contractions in the presence of the drug. p<0.05 are highlighted in bold. (DOC) [file pone.0019597.s005.doc]

|  | Length of WL contraction | | | Speed of WL contractions | | | Length of stationary contractions | | |
| --- | --- | --- | --- | --- | --- | --- | --- | --- | --- |
|  | mm | N | mm | mm | mm | P | mm | N | P |
| Control | 47.8 ± 3.0 | 9 | 4.8 ± 0.5 | 4.8 ± 0.5 | 4.8 ± 0.5 |  | 16.1 ± 1.8 | 9 |  |
| TRAM34 | 46.6 ± 3.2 | 10 | 4.9 ± 0.2 | 4.9 ± 0.2 | 4.9 ± 0.2 | 0.839 | 13.7 ± 0.9 | 10 | 0.220 |
| Clotrimazole | 46.9 ± 2.4 | 10 | 3.7 ± 0.3 | 3.7 ± 0.3 | 3.7 ± 0.3 | 0.977 | 10.9 ± 0.8 | 10 | **0.014** |
| NAN-190 | 48.7 ± 1.7 | 7 | 4.2 ± 0.3 | 4.2 ± 0.3 | 4.2 ± 0.3 | 0.114 | 16.8 ± 1.7 | 7 | 0.788 |
| WAY-100135 | 54.7 ± 5.0 | 6 | 4.0 ± 0.2 | 4.0 ± 0.2 | 4.0 ± 0.2 | 0.592 | 17.1 ± 2.2 | 6 | 0.729 |
