# Supplementary material for: Multiple Neural Oscillators and Muscle Feedback Are Required for the Intestinal Fed State Motor Program
Source: PLoS One. 2011 May 5;6(5):e19597. doi: 10.1371/journal.pone.0019597 (PMC3088688; doi:10.1371/journal.pone.0019597)
Supplement: Table S6 — This table provides the measurement data for properties of orally and anally directed short-length propagating (SL) contractions in the presence of the drug. p<0.05 are highlighted in bold. (DOC) [file pone.0019597.s006.doc]

|  | Length of anal SL contractions | | | Speed of anal SL contractions | | | Length of oral SL contractions | | | Speed of oral SL contractions | | |
| --- | --- | --- | --- | --- | --- | --- | --- | --- | --- | --- | --- | --- |
|  | mm | N | P | mm s^-1^ | N | P | mm | N | P | mm s^-1^ | N | P |
| Control | 16.1 ± 1.8 | 9 |  | 7.8 ± 0.9 | 9 |  | 9.5 ± 1.2 | 9 |  | 6.5 ± 1.0 | 9 |  |
| TRAM34 | 13.7 ± 0.9 | 10 | 0.220 | 7.7 ± 0.4 | 10 | 0.972 | 9.9 ± 0.6 | 10 | 0.761 | 9.0 ± 0.7 | 10 | 0.057 |
| Clotrimazole | 10.9 ± 0.8 | 10 | **0.014** | 7.6 ± 0.5 | 10 | 0.819 | 7.9 ± 0.6 | 10 | 0.224 | 5.3 ± 0.8 | 10 | 0.349 |
| NAN-190 | 16.8 ± 1.7 | 7 | 0.788 | 9.8 ± 1.0 | 7 | 0.150 | 8.9 ± 0.7 | 7 | 0.682 | 7.1 ± 0.7 | 7 | 0.639 |
| WAY-100135 | 17.1 ± 2.2 | 6 | 0.729 | 7.1 ± 0.4 | 6 | 0.567 | 8.3 ± 0.6 | 6 | 0.442 | 6.2 ± 0.5 | 6 | 0.812 |
