# Supplementary material for: Multiple Neural Oscillators and Muscle Feedback Are Required for the Intestinal Fed State Motor Program
Source: PLoS One. 2011 May 5;6(5):e19597. doi: 10.1371/journal.pone.0019597 (PMC3088688; doi:10.1371/journal.pone.0019597)
Supplement: Table S7 — This table provides the data on the effects of drugs on bursts of contractions. p<0.05 are highlighted in bold. (DOC) [file pone.0019597.s007.doc]

|  | Number of contractions in a burst | | | Frequency of contractions within a burst | | | Frequency of bursts | | |
| --- | --- | --- | --- | --- | --- | --- | --- | --- | --- |
|  | burst^-1^ | N | P | contractions min^-1^ | N | P | bursts min^-1^ | N | P |
| Control | 3.0 ± 0.4 | 9 |  | 11.6 ± 2.4 | 9 |  | 0.5 ± 0.2 | 9 |  |
| TRAM34 | 3.5 ± 0.1 | 10 | 0.631 | 17.5 ± 0.6 | 10 | 0.050 | 1.2 ± 0.2 | 10 | **0.034** |
| Clotrimazole | 3.6 ± 0.1 | 10 | 0.347 | 15.7 ± 1.0 | 10 | 0.265 | 2.6 ± 0.7 | 10 | **0.020** |
| NAN-190 | 2.3 ± 0.3 | 7 | 0.345 | 10.5 ± 3.7 | 7 | 0.808 | 0.5 ± 0.2 | 7 | 0.968 |
| WAY-100135 | 3.1 ± 0.1 | 6 | 0.913 | 11.2 ± 2.8 | 6 | 0.911 | 0.3 ± 0.1 | 6 | 0.581 |
