# Supplementary material for: Multiple Neural Oscillators and Muscle Feedback Are Required for the Intestinal Fed State Motor Program
Source: PLoS One. 2011 May 5;6(5):e19597. doi: 10.1371/journal.pone.0019597 (PMC3088688; doi:10.1371/journal.pone.0019597)
Supplement: Table S8 — Drug effects on the duration of active and quiescent episodes. p<0.05 are highlighted in bold. (DOC) [file pone.0019597.s008.doc]

|  | Duration of quiescent periods | | | Duration of active episodes | | |
| --- | --- | --- | --- | --- | --- | --- |
|  | s | N | P | s | N | P |
| Control | 48 ± 7 | 9 |  | 38 ± 12 | 9 |  |
| TRAM34 | 64 ± 12 | 10 | 0.284 | 141 ± 44 | 10 | **0.049** |
| Clotrimazole | 52 ± 10 | 10 | 0.814 | 245 ± 80 | 10 | **0.028** |
| NAN-190 | 35 ± 3 | 7 | 0.120 | 114 ± 80 | 7 | 0.300 |
| WAY-100135 | 39 ± 3 | 6 | 0.307 | 58 ± 22 | 6 | 0.397 |
